# New Surgical Model for Bone–Muscle Injury Reveals Age and Gender-Related Healing Patterns in the 5 Lipoxygenase (5LO) Knockout Mouse

**DOI:** 10.3389/fendo.2020.00484

**Published:** 2020-08-11

**Authors:** Claudia Cristina Biguetti, Maira Cristina Rondina Couto, Ana Claudia Rodrigues Silva, João Vitor Tadashi Cosin Shindo, Vinicius Mateus Rosa, André Luis Shinohara, Jesus Carlos Andreo, Marco Antonio Hungaro Duarte, Zhiying Wang, Marco Brotto, Mariza Akemi Matsumoto

**Affiliations:** ^1^Department of Basic Sciences, School of Dentistry, São Paulo State University (UNESP), Araçatuba, Brazil; ^2^Bone-Muscle Research Center, College of Nursing and Health Innovation, University of Texas at Arlington, Arlington, TX, United States; ^3^Department of Health Sciences, Universidade Do Sagrado Coração, Bauru, Brazil; ^4^Bauru School of Dentistry, University of São Paulo, FOB-USP, São Paulo, Brazil

**Keywords:** 5 lipoxygenase, bone, lipid mediators, aging, muscle, tissue healing

## Abstract

Signaling lipid mediators released from 5 lipoxygenase (5LO) pathways influence both bone and muscle cells, interfering in their proliferation and differentiation capacities. A major limitation to studying inflammatory signaling pathways in bone and muscle healing is the inadequacy of available animal models. We developed a surgical injury model in the *vastus lateralis* (VL) muscle and femur in 129/SvEv littermates mice to study simultaneous musculoskeletal (MSK) healing in male and female, young (3 months) and aged (18 months) WT mice compared to mice lacking 5LO (5LOKO). MSK defects were surgically created using a 1-mm punch device in the VA muscle followed by a 0.5-mm round defect in the femur. After days 7 and 14 post-surgery, the specimens were removed for microtomography (microCT), histopathology, and immunohistochemistry analyses. In addition, non-injured control skeletal muscles along with femur and L5 vertebrae were analyzed. Bones were microCT phenotyped, revealing that aged female WT mice presented reduced BV/TV and trabecular parameters compared to aged males and aged female 5LOKO mice. Skeletal muscles underwent a customized targeted lipidomics investigation for profiling and quantification of lipid signaling mediators (LMs), evidencing age, and gender related-differences in aged female 5LOKO mice compared to matched WT. Histological analysis revealed a suitable bone-healing process with osteoid deposition at day 7 post-surgery, followed by woven bone at day 14 post-surgery, observed in all young mice. Aged WT females displayed increased inflammatory response at day 7 post-surgery, delayed bone matrix maturation, and increased TRAP immunolabeling at day 14 post-surgery compared to 5LOKO females. Skeletal muscles of aged animals showed higher levels of inflammation in comparison to young controls at day 14 post-surgery; however, inflammatory process was attenuated in aged 5LOKO mice compared to aged WT. In conclusion, this new model shows that MSK healing is influenced by age, gender, and the 5LO pathway, which might serve as a potential target to investigate therapeutic interventions and age-related MSK diseases. Our new model is suitable for bone–muscle crosstalk studies.

## Introduction

Investigation of bone and muscle homeostasis has in recent years expanded to biochemical crosstalk via the secretion of different molecules, such as signaling lipid mediators (LMs) derived from essential polyunsaturated fatty acids (PUFA), with potential implications on aging, inflammation, and tissue healing (1–4). In this context, although skeletal muscle possesses great plasticity in response to physiologic stimuli (5, 6), its capacity for adaptation/regeneration is dependent on many different factors, such as the nature and extent of the stimulus (7), viability of satellite cells, aging, and inflammation (7–9). Indeed, higher inflammatory marker levels in elderly populations are directly associated with loss of muscle mass and strength (10), as well with the reduced capacity for bone healing, decreasing in the viability of osteoprogenitor cells (11). As observed in mice, aging also causes changes in skeletal muscle in a variable range of LM (12), such as AA-derived eicosanoids, eicosapentaenoic acid (EPA), as well as Omega 3 (ω-3) PUFA derivatives [e.g., docosahexaenoic acid [DHA]], in an age- and gender-dependent manner. In the face of these multiple factors (inflammation, aging, and gender), healing outcomes on the MSK system can vary from complete tissue regeneration to fibrosis, affecting the functional recovery of these tissues with important clinical implications (11). However, investigations are still ongoing on the specific cellular and molecular factors involved in the interconnected and interdependent healing of bone and muscle.

Due to the close anatomical relationship of bone and muscle, traumatic bone injuries (open bone fractures or surgical trauma) involve damage to the adjacent muscles, requiring simultaneous tissue healing of both compartments (1, 2, 13). Skeletal muscle assists bone healing, not only when it is used as a flap (or a second periosteum) for improving bone defect vascularization (14–16), but also the osteogenic potential of satellite cells (13). *In vitro* studies have suggested that osteocytes can enhance *in vitro* myogenesis and *ex vivo* muscle contractility by different mechanisms, such as through the Wnt/β-Catenin pathway (3) and the secretion of prostaglandin E_2_ (PGE_2_) key LM, which has a positive impact in the myogenic differentiation of primary mouse myoblasts/myotubes (4).

Skeletal muscle is mainly composed by muscle fibers surrounded by myogenic progenitors (satellite cells) with the capacity to proliferate and induce new fiber formation to restore injured tissue, followed by the upregulation and expression of Myoblast Determination Protein 1 (MyoD), as demonstrated in murine models of muscle regeneration (7, 17). MyoD is a quintessential protein in mammals that belongs to the Muscle Regulatory Family of proteins and is necessary for myoblast differentiation into myotubes (18). While new myotubes are formed, other injured muscle fibers undergo to degeneration/atrophy due to injuries followed by the upregulation and expression of Muscle Ring Finger-1 (MuRF1) (7, 17, 19). MuRF1 belongs to the family of ubiquitin ligases, is proposed to trigger muscle protein degradation via ubiquitination, serving as a marker for fiber degeneration/atrophy (19). MyoD and MuRF1 are both suitable markers for models of muscle regeneration/remodeling in skeletal muscle post-surgery. At the same time, bone has mesenchymal cell-derived osteoblasts, which can transfer from a quiescent stage into rapid proliferation and differentiation after certain stimuli and expression of Runt-related Transcription Factor-2 (Runx-2) (20). After new bone deposition, hematopoietic-derived osteoclasts regulated by osteoblasts, and inflammatory mediators contribute to bone maturation by a coordinated resorption of new bone matrix, followed by expression of different markers, such as Tartrate Resistant Acid Phosphatase (TRAP) (21).

Apart from the specificity of each cell type involved in skeletal muscle and/or bone regeneration, general tissue healing post-injury requires an initial and transient inflammatory phase, with release of several cytokines and inflammatory mediators, such as AA-derived signaling lipid mediators released from ciclooxigenase-2 (COX2) and 5-Lipoxygenase (5LO) pathways (22–24). These processes inherent to the inflammatory response are essential in directing vascular events and leukocytes migration, through the release of PGs and LTs (LTB4 and/or CysLTs), respectively (25, 26). In addition, evidence from other studies have also supported a crucial modulatory role of COXs and 5LO pathways and their products, PGs and LTs, on bone and muscle cells in different models of tissue repair/regeneration (4, 27–31).

While COX2 seems to affect bone healing in a positive manner by inducing new bone formation and angiogenesis (27, 32, 33), the 5LO pathway and its final products are supposed to contribute to osteoclastic differentiation and consequent bone resorption (29, 34–36) and inhibited bone formation *in vitro* (37). Male mice genetically deficient (knockout) for COX2 expression (COX2 KO mice), presented delayed bone formation in femur fractures, but with significant rescue after periosteal injection of the prostaglandin E2 receptor 4 (EP4) agonist (33). On the other hand, male young mice lacking 5LO expression presented increased cortical thickness (36) and accelerated fracture healing in endochondral bones (28). Previous studies comparing 129 SvEv WT with homozygous KO for 5LO have also shown a relevant role of 5LO activity in periodontal inflammation and bone resorption (35, 38). Furthermore, the pharmacological inhibition of 5LO (39) or antagonism of CysLT1-receptor also enhanced endochondral bone healing in rats (40). In skeletal muscle, LTB4 contributes to muscle regeneration by enhancing the proliferation and differentiation of satellite cells (41). *In vitro* studies revealed that the 5LO pathway is one of the major sources of extracellular ROS release in skeletal muscle (42). Significant upregulation of LTB4 pathway has been particularly found in muscle tissue in chronic inflammatory conditions (polymyositis or dermatomyositis) and seems to be associated with muscle weakness (43). However, the role of 5LO on muscle healing remains elusive, and no previous studies have addressed the effects of 5LO inhibition or its complete deletion in animal models.

Finally, it is important to mention that 5LO activity measured by the amount of LTs are significantly higher in females than in males, in humans (44, 45), and rodents (46–48). These differences in gender generally associate with a higher incidence and severity of inflammatory chronic conditions and autoimmune diseases in females than males, such as rheumatoid arthritis and asthma, and positively associate with increased levels of LTs (49). Thus, it is reasonable to hypothesize that gender is an important factor that can modulate the action of lipid mediators on the MSK system-healing capacity.

Therefore, efforts on development/characterization of animal models of simultaneous bone–muscle healing might support a comprehensive understanding on the role of lipid mediators in this process to provide insights that could lead to the identification of potential targets for the treatment and/or new interventions (e.g., specific diets/exercises) of MSK injuries. Our primary objective was to develop a new surgical mouse model that better represents the pathophysiological conditions for the simultaneous injury of bone and muscle without major catastrophic failure. As a secondary objective, we then utilized this model to investigate healing patterns during aging. We further studied the influence of gender and 5LO using the 5LOKO mouse. Our key findings revealed that aging delayed both muscle and bone healing in mice, with bones recovering faster than muscles, maybe indicating a role of muscles, to act as bone sentinels. We observed the major negative effects in aged WT females. Importantly, despite the aging negative effects, 5LOKO aged females presented an improved skeletal phenotype and healing outcomes compared to the young WT mice.

## Materials and Methods

### Animals

In this study, we utilized 129/SvEv wild type (WT) and 5LO (homozygous knockout for 5LO^tm1Fun^, designated here as 5LOKO). Littermate controls 129/SvEv and 5LOKO mice were used as previously described by other studies using this strain of 5LOKO mice (35, 38) and from the same source (38). Mice were obtained from Central Animal Facility for Special Mice of the School of Medicine of Ribeirão Preto—University of São Paulo, Brazil (CCCE-FMRP-USP) and were housed in the Central Animal Facility of Universidade Sagrado Coração (USC), Bauru, São Paulo, Brazil, under the approval of IACUC protocol #9589271017 (from Institutional Ethic Committee on Animal Use) and following the normative regulations provided by the National Council for the Control of Animal Experimentation in Brazil (CONCEA). Our design included four experimental groups, each containing 20 mice: 3-month old (young) males and females, and 18-month old (aged) males and females. The mice received sterile water and sterile standard solid mice chow (Nuvital, Curitiba, PR, Brazil) *ad libitum* and were housed at temperature-controlled rooms (22–25°C). We conducted estrus cycle analysis (Toluine blue O vaginal smears) to determine post-menopausal stage in all aged female mice (50, 51). All animal procedures were cared for in accordance with the CONCEA and following the Guide for the Care and Use of Laboratory Animals of the National Institutes of Health (Institute of Laboratory Animal Resources (U.S.), as well as the ARRIVE guidelines recommendations (52). Experimental groups for bone–muscle surgical injury model were comprised of five animals per group/time point (7 and 14 days) and used for microtomography (microCT), histological, and immunohistochemical analysis. Prior and during the experimental protocol, animals from each group were allocated in groups of five mice per cage and the cages were codified by the researcher supervisor and technician in order to minimize bias. Naïve bones (left femur and L5 vertebrae) were used for skeletal phenotyping by microCT analysis. One 129SvEv WT aged and one young female were additionally used for pilot studies for the surgical model. Naïve gastrocnemius muscles were collected for lipidomics analyses at the Bone–Muscle Research Center, https://www.uta.edu/conhi/research/bmrc/index.php, University of Texas–Arlington.

### Experimental Protocol for Surgical Muscle and Bone Injury

We developed a new surgical model for the simultaneous injury of bone and muscle to simulate conditions of trauma and/or surgeries in a controlled and reproducible manner, where both tissues are damaged, but without catastrophic damage, such as an injury that occurs in automobile accidents or with military personnel. Mice were anesthetized with intraperitoneal injection of 80 mg/kg ketamine chloride (Dopalen, Agribrands Brasil, Paulínia, SP, Brazil) and 160 mg/kg xylazine chloride (Anasedan, Agribrands Brasil, Paulínia, SP, Brazil). Additional local anesthesia was provided by using 5 μL of intramuscular injection of Mepivacaine Hydrochloride 2% with Epinephrine 1:100,000 (Mepiadre®, DFL, Rio de Janeiro, Brazil) vasoconstrictor, to provide hemostasis and additional comfort in the first hours after surgery. All animals were measured in relation to body weight (BW) and rostrocaudal (RC) length before the experimental procedures. Then, mice were positioned in lateral decubitus under a stereomicroscope providing 5× magnification (DF Vasconcellos, São Paulo, SP, Brazil). The right lower limb was gently shaved, disinfected with polyvinylpyrrolidone prior to a 10-mm vertical incision to expose the Vastus Lateralis (VL) underlying muscle. A 1-mm micro-punch device for rodents (Härte Instrumentos Cirúrgicos, Ribeirao Preto, Brazil) was used to create a muscle defect to a depth of 1.5 mm until it reached the femoral bone. To produce a subjacent monocortical bone defect in the femur, midshaft femoral bone was drilled using a 0.50-mm pilot drill (NTI-Kahla GmbH Rotary Dental Instruments, Kahla, Thüringen, Germany) to a maximum depth of 1 mm at 600 rpm, using a surgical motor (NSK-Nakanishi International, Kanuma, Tochigi, Japan). Bone defects were created under cold saline solution irrigation to avoid thermal necrosis in bone and surrounding tissues. Only subcutaneous tissue (not muscle) was sutured with 5–0 silk suture (Ethicon, Johnson & Johnson, São Paulo, Brazil). All groups of animals were submitted to surgery, and the actual surgical procedure (creation of defects) was performed by a single trained, expert surgeon (CCB), who was blinded at this point of the experiment. No antibiotics and anti-inflammatory drugs were administered to the animals after the MSK-injury so as not to interfere with experimental design. At the first 72 h after surgery, the feed was supplied from the bottom compartment of the cage to prevent, but not impede, the recovering mice from getting up in the cage, which could result in additional unintended damage. The suture naturally fell off 3–4 days post-surgery when the skin was clinically healed. Mice were provided sterile water *ad libitum* and were fed with sterile standard solid mice chow (Nuvital, Curitiba, PR, Brazil) throughout all experimental periods of this study.

### Sample Collection

At the end of experimental time points, mice were euthanized for sample collection. The VL muscle was dissected from the quadriceps femoris muscles, embedded in optimal cutting temperature compound (OCT) (Tissue-Tek, Sakura Finetek, Torrance, CA, USA) and immediately frozen in liquid nitrogen for cryostat sectioning. Gastrocnemius muscle samples were collected and immediately frozen in liquid nitrogen for targeted quantification of LMs via lipidomics, as previously described (12). Injured femurs, as well as femur controls and L5 vertebrae, were fixed in phosphate buffered saline (PBS) buffered formalin (10%) solution (pH 7.2) for 48 h at room temperature. Bone specimens were washed overnight in running water and maintained in 70% hydrous ethanol for microCT scanning. After microCT scanning, bone specimens were decalcified in 4.13% EDTA (pH 7.2) following histological processing protocols, as previously described (53).

### Micro CT

Bone specimens (injured, control femurs, and L5 vertebrae) were scanned for qualitative and quantitative analyses by microCT. Controls used for bone phenotyping (10 biological replicates) from each group were pooled (from 7 to 14 days), since they represented controls. Five biological replicates of injured femurs were used from each group. First, specimens were rehydrated in saline solution for 10 min before scanning. Sample scanning was performed by using a Skyscan 1174 System (Skyscan, Kontich, Belgium) at 50 kV, 800 μA, with a 0.5-mm aluminum filter, 180 degrees of rotation and exposure range of 1 degree and a 14-μm-pixel-size resolution, as previously described (21). Briefly, projections were first reconstructed using the NRecon software (Bruker microCT, Kontich, Belgium), realigned in Data Viewer for 2D images. Tridimensional images were obtained by using CTVox (Bruker microCT, Kontich, Belgium). Quantitative parameters were assessed using CTAn (Bruker microCT, Kontich, Belgium), based on previous guidelines and recommendations (54), as well as similar regions of interest (ROI) for L5 vertebrae and femur analysis (55–57). For skeletal phenotype, L5- vertebral body was evaluated considering a ROI, 1.5 mm in diameter and 3 mm in length. Femur was analyzed considering two regions: a 2-mm length of the cortical of mid-diaphysis and a 1.5-mm length of the cancellous compartment of distal metaphysis. Evaluated parameters for skeletal phenotype comprised bone volume (BV, mm^3^), fraction of bone volume [Bone Volume/Tissue Volume (BV/TV), %], trabecular number (Tb.N), trabecular thickness (Tb.Th), and trabecular separation (Tb.Sp). Cross-sectional volume (mm^3^) was evaluated only for the cortical mid-diaphysis of femur. Bone defects were evaluated for BV/TV (%) by using a cylindrical ROI, 0.5 mm in diameter and 0.5 mm in depth, considering the size of monortical defect.

### Histological Processing for Injured Bone

After microCT scanning, femur samples containing the region of defect were washed and immersed in buffered 4.13% EDTA (pH 7.2) for decalcification for 3 weeks. Then, specimens were processed for histological embedding in paraffin blocks. Semi-serial 5-μm histological transversal slices were obtained from the central area of the bone defect and were used for Hematoxylin and Eosin (H&E), modified Goldner's Trichrome/Alcian Blue (58), and Picrosirius red staining for birefringence and immunohistochemistry for 5LO, Runx2, and TRAP.

### Histological Processing for Injured Muscles

Muscle samples were processed as previously described (59, 60). VL muscles were embedded in OCT and sectioned at temperature of −20°C (Leica, CM 1850, Nussloch, Germany). Eight semi-serial 8-μm transversal sections were obtained from each specimen separated by at least 40 μm, comprising the area of defect and adjacent areas. Histological sections from days 7- to 14 post-surgery were used for H&E staining. Histological sections from 7 days post-surgery were used for immunohistochemistry for MuRF1 and MyoD.

### Histopathological Analysis of Bone and Muscle Healing

Bone and muscle samples stained with H&E were used for histopathological analysis by two blinded examiners (CCB and MAM). Bone healing was evaluated considering the presence of inflammatory infiltration, connective tissue, cartilage, newly formed bone (containing new osteoblasts and osteocytes differentiation), osteoclasts, and blood vessels in remodeling areas. Modified Goldner's Trichrome/Alcian Blue was also used as a complementary approach to identify newly formed bone, cartilage, and connective tissue. Five biological replicates from muscle samples were also used for histomorphometric analysis. The histological region for healing comprised 1 mm^2^ in the central area of muscle defect, as well the adjacent region of central damage, for evaluation of cross-sectional area (CSA) of fibers. CSA measurement was performed with two technical replicates from each animal, from which six photomicrographs at 100× magnification (technical replicates) were captured and evaluated using the software SigmaScan Pro 5.0 (Systat Software Inc., Chicago, USA). Histomorphometric parameters included inflammatory infiltrate in the area of injury and fibers with centralized myonuclei surrounding the region of the defect. We used histological sections (three technical replicates) from five animals (biological replicates) per group. Quantification of histological parameters was performed using four random histological fields from each histological section. Histological fields were captured at 100× using an oil immersion objective (Carl Zeiss Jena GmbH, Jena, Germany). A grid image was superimposed on each histological field containing a total of 100 points in a quadrangular area by using the ImageJ software (Version 1.51, National Institutes of Health, Bethesda, Maryland, USA), as previously described (53). Only the points coincident (considered as the intersection point of the vertical and horizontal lines) with the histological parameters were considered, and the total number of points was obtained to calculate the area density for each healing component in each section. Results were presented as means ± SDs of the area density for each bone and muscle healing parameter.

### Birefringence Analysis for Collagenous Content in Bone Defects

Quality and quantity of bone matrix deposition was performed using the Picrosirius-polarization method and birefringence analysis. Two histological fields (two biological replicates) of each femur defects stained with Picrosirius Red were captured with a 10× objective under polarizing lens coupled to a binocular inverted microscope (Leica DM IRB/E) and analyzed as previously described (21, 61). Green birefringence color indicates thin fibers; yellow and red colors at birefringence analysis indicate thick and organized collagen fibers (21, 58). Briefly, spectra of green, yellow, and red colors were defined by RGB values, and the quantity of pixels-squared was calculated for each field by using the AxioVision 4.8 software (CarlZeiss). After calculations of each spectrum fiber, total area was also calculated by the sum of each color spectrum. Means and standard deviation (SD) considering the two technical replicates (histological fields) and five biological replicates (number of animals per group) were calculated for each group, considering strain, gender and age.

### Immunohistochemistry

Histological sections from femur defects were used for individual immune detection of TRAP (sc30832), Runx2 (sc8566), and 5LO (sc136195). Muscle samples were used for individual immune detection of MuRF-1 (sc398608) and MyoD (sc377460). All primary antibodies were purchased from Santa Cruz Biotechnology (Santa Cruz Biotechnology, Santa Cruz, CA, USA). Paraffin-embedded histological sections were rehydrated, and antigen retrieval was performed by boiling the slides in 10 mM sodium citrate buffer (pH 6) for 30 min at 100°C. Sections were pre-incubated with 3% Hydrogeroxidase Block (Spring Bioscience Corporation, CA, USA) and then incubated with 7% Non-fat Dry Milk to block serum proteins. Frozen muscles samples were incubated in ice-cold acetone prior to incubation with horse serum for protein blocking. Anti-TRAP, anti-Runx2, Anti-MuRF1, and Anti-MyoD primary antibodies were diluted at 1:100, while 5LO was diluted in 1:200 in diluent solution and then incubated for 1 h at room temperature. Goat-On-Rodent HRP-Polymer PromARM (cat # GHP516G, Biocare Medical, Pacheco, CA) was used as a detection method for TRAP and Runx-2, and sections were incubated for 40 min at room temperature following manufacturer instructions. Anti-Mouse HRP-Polymer made in goat (ImmPRESS, Vector Laboratories, San Diego, CA) was used as a detection method for 5LO, MuRF-1, and MyoD. The identification of antigen–antibody reaction was performed using 3-3′-diaminobenzidine (DAB). Bone samples labeled for TRAP, Runx-2, and 5LO were counterstained with Mayer's hematoxylin. Muscle samples were left without counterstaining to be used for optical density quantification. Negative controls were performed by using only diluents solution instead of primary antibody.

### Quantification of Immunohistochemical Markers

Quantification of all markers was performed by using four histological fields from each histological section captured, using a 100× oil immersion objective (Carl Zeiss Jena GmbH, Jena, Germany). Quantification of TRAP was performed at the 14-day time point; most TRAP+ cells were found in areas of bone remodeling. Runx2, MuRF-1, and MyoD labeling were analyzed at the 7-day time point. Pixels quantification was performed with Aperio Image Scope v. 12.3.3 (Leica Biosystems, Buffalo Grove, USA), used for immunohistochemical analysis in pathology (62). Images were analyzed by choosing the algorithm Positive Pixel Count in Aperio Image Scope (Leica Biosystems, Buffalo Grove, USA). Subsequently, all positive pixels were calculated, and the results were expressed as Positivity (number of positive pixels/number total). Negative controls tissues were used for confirmation of negative labeling and detection. Results were presented as the means and SD for each marker.

### Targeted Lipidomics

Targeted lipidomics was performed following our previously published quantification method (12) with some modifications. Briefly, aliquoted frozen muscle tissue (50–100 mg) was applied for LC-MS/MS-based lipidomic analysis. All components of LC-MS/MS system are from Shimadzu Scientific Instruments, Inc. (Columbia, MD). The LC system was equipped with four pumps (Pump A/B: LC-30AD, Pump C/D: LC-20AD XR), a SIL-30AC autosampler (AS), and a CTO-30A column oven containing a two-channel, six-port switching valve. The LC separation was conducted on a C8 column (Ultra C8, 150 × 2.1 mm, 3 μm, RESTEK, Manchaca, TX) along with a Halo guard column (Optimize Technologies, Oregon City, OR). The MS/MS analysis was performed on Shimadzu LCMS-8050 triple quadrupole mass spectrometer. The instrument was operated and optimized under both positive and negative electrospray and multiple reaction monitoring modes (± ESI MRM). Standard lipid mediators and corresponding isotope-labeled lipid mediator internal standards (IS) were purchased from Cayman Chemical Co. (Ann Arbor, MI). All analyses and data processing were completed on Shimadzu LabSolutions V5.91 software (Columbia, MD). Four total bioactive lipids [EPA, DHA, AA, 11,12-epoxyeicosatrienoic acid [11,12-EET], and PGE_2_] were quantified from muscle samples in this study. Results were presented quantity of each lipid in muscle (pg/mg muscle).

### Statistical Analysis

Quantitative data were first analyzed for distribution of normality using Shapiro-Wilk normality test. Possible outliers were identified using the ROUT method, using the maximum Q of 1% for False Discovery Rate (FDR) (63). The effect of age and/or gender and/or genotype for quantitative parameters were analyzed by two-way ANOVA followed by Bonferroni correction. Values of *p* < 0.05 were considered statistically significant.

## Results

### Differences in Gross Anatomy and Skeletal Phenotype of 5LOKO Aged Mice

**Figure 1:**
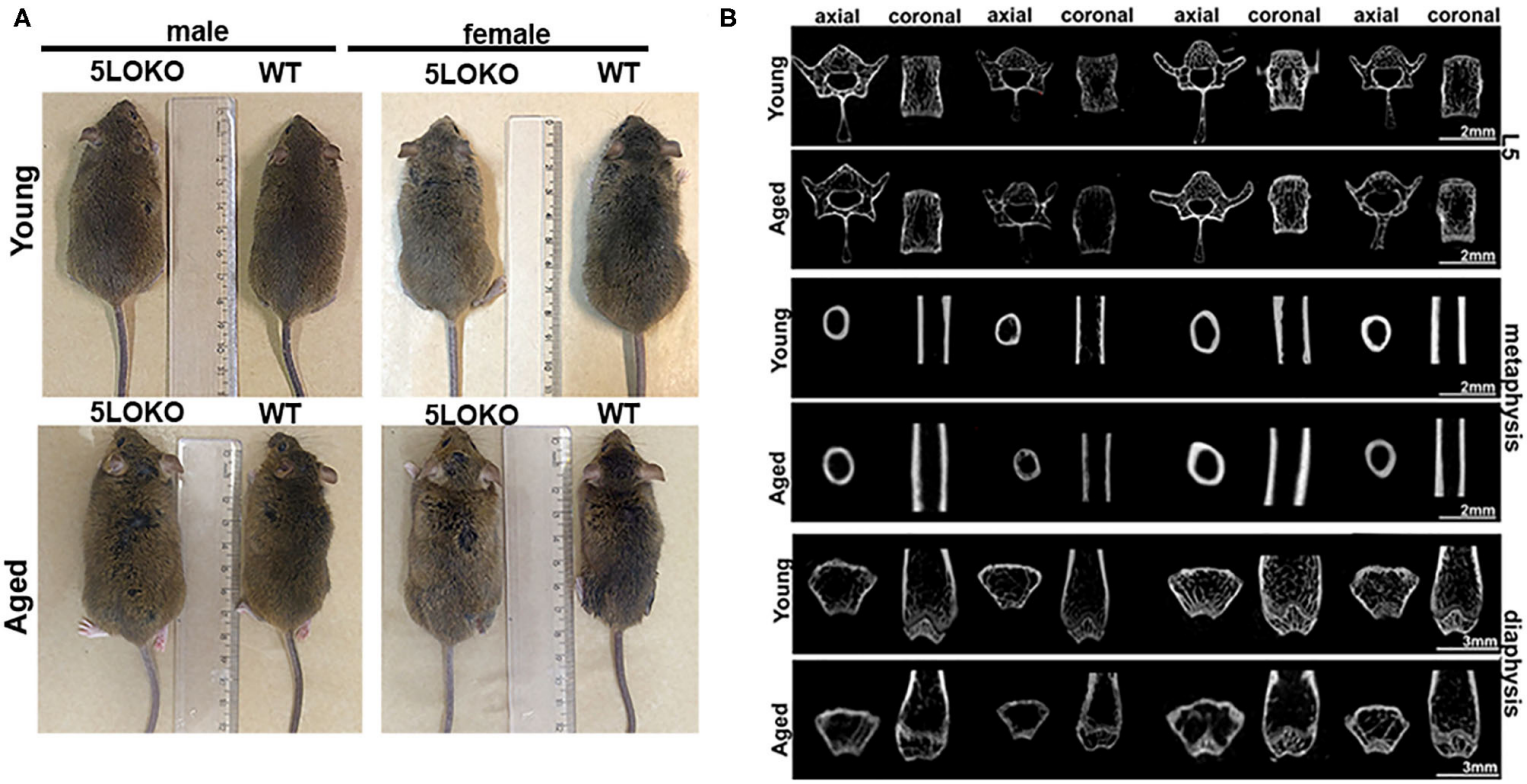
Macroscopic anatomy and skeletal phenotype of young and aged WT and 5LOKO mice: **(A)** Male and female, young (3 months old) and aged (18 months old) 129/SvEv WT and 5LOKO mice. **(B)** Axial and Coronal sections of L5 vertebrae, femur distal metaphysis, and mid-diaphysis. Images were obtained after scanning in Skyscan 1174 System (Skyscan, Kontich, Belgium). Projections were first reconstructed using the NRecon software and realigned in Data Viewer (Bruker, Kontich, Belgium) for 2D images.

First, we investigated the impact of aging on body weight, RC length, and microtomographic parameters for skeletal phenotype in male and female 129/SvEv WT and 5LOKO mice (Figure 1, Table 1). Statistically significant differences for the body weight (grams) and RC length (cm) were found between genders in WT groups: higher values for young and aged male compared to female mice. Also, young 5LOKO male presented increased body weight compared to females, and aged 5LOKO male mice presented increased RC length compared to females. Considering differences between genotypes, aged 5LOKO males showed higher RC length compared to aged controls, while no differences were found between aged or young WT vs. 5LOKO females considering these parameters.

**Table 1:** Macroscopic features and skeletal phenotyping in WT vs. 5LOKO mice.

**Parameters**	**Sites/Groups**	**Y WT ♂**	**Y WT ♀**	**A WT ♂**	**A WT ♀**	**Y KO ♂**	**Y KO ♀**	**A KO ♂**	**A KO ♀**
BW (grams)	–	27.97 ± 3.22^&^	22.54 ± 2.36^&^	29.06 ± 3.22^&^	23.96 ± 3.58^&^	25.90 ± 3.07^&^	20.61 ± 2.00^&^^*^	29.04 ± 2.59	26.07 ± 2.49^*^
RC (cm)	–	9.07 ± 0.27^&^	8.43 ± 0.30^&^	8.66 ± 0.30^#^	8.51 ± 0.47	9.00 ± 0.23	8.50 ± 0.16	9.37 ± 0.49^&^^#^	8.79 ± 0.42^&^
BV (mm^3^)	Femur diaphysis	1.70 ± 0.09	1.90 ± 0.80	2.22 ± 0.31	1.62 ± 0.32	1.98 ± 0.22	1.59 ± 0.41	1.70 ± 0.26	1.74 ± 0.64
BV/TV (%)	Femur metaphysis	16.98 ± 5.12	23.99 ± 5.81^*^	19.08 ± 2.87	12.30 ± 2.20^*^^#^	28.42 ± 7.00	27.91 ± 54.98	27.30 ± 3.92	21.00 ± 4.19^#^
	L5 vertebrae	35.88 ± 4.87	44.16 ± 4.35^*^	33.68 ± 7.45	25.62 ± 6.65^*^^#^	40.08 ± 3.28	34.15 ± 5.25	42.63 ± 4.41	42.27 ± 7.06^#^
Tb.Th (mm)	Femur metaphysis	0.18 ± 0.01	0.19 ± 0.02	0.17 ± 0.02	0.16 ± 0.03	0.19 ± 0.03	0.19 ± 0.04	0.21 ± 0.06	0.21 ± 0.02
	L5 vertebrae	0.09 ± 0.01	0.10 ± 0.01	0.10 ± 0.02	0.10 ± 0.03	0.10 ± 0.01	0.13 ± 0.02	0.12 ± 0.01	0.17 ± 0.03
Tb.Sp (mm)	Femur metaphysis	0.46 ± 0.18	0.33 ± 0.07^*^	0.53 ± 0.12	0.86 ± 0.39^*^	0.33 ± 0.09	0.30 ± 0.06^*^	0.31 ± 0.109^&^	0.70 ± 0.07^*^^&^
	L5 vertebrae	0.29 ± 0.03	0.24 ± 0.02	0.34 ± 0.06^#^	0.32 ± 0.07^#^	0.20 ± 0.03	0.21 ± 0.04	0.21 ± 0.01^#^	0.17 ± 0.03^#^
Tb.N (1/mm)	Femur metaphysis	1.05 ± 0.16	1.18 ± 0.30	0.99 ± 0.15	0.61 ± 0.11	1.45 ± 0.12	1.67 ± 0.43^*^	1.53 ± 0.32	0.85 ± 0.26^*^
	L5 vertebrae	3.22 ± 0.25	3.51 ± 0.34^*^	2.60 ± 0.66^&^	1.28 ± 0.36^*^^#&^	3.96 ± 0.39	3.15 ± 0.18	3.07 ± 0.26	2.18 ± 0.28^#^

*Results are presented as the means (± SD) for each parameter*.

In the comparison between columns, symbol

* indicates comparison between columns: the effect of age (Y vs. A) in groups of same genotype and gender (e.g., Y ♀ WT vs. A ♀ WT); symbol

# indicates the effect of different genotypes (WT vs. KO) in groups of same age and gender (e.g., Y ♀ WT vs. Y ♀ KO); and symbol

& *indicates the effect of gender (♂ vs. ♀) in the same genotype and age (e.g., Y ♀ WT vs. Y ♂ WT). Statistically significant differences are indicated between groups with equal symbols (p < 0.05). Y = 3 months; A = 18 months. BW, Body weight; RC, rostrocaudal length*.

We evaluated the cortical diaphysis of femur (BV) and morphological parameters in cancellous compartments of femur (distal metaphysis) and the L5-vertebral body. No statistical differences were found in the cortical diaphysis of femur, considering age, gender, or genotype (Table 1); although qualitative differences were observed in the transversal sections of femur diaphysis of aged WT females compared to aged WT males and aged 5LOKO females. Aged WT and aged 5LOKO females displayed increased trabecular spacing (Tb.Sp) in femur metaphysis compared to their young controls (*p* < 0.05), but presented no differences comparing both genotypes. The effect of age led to a reduced BV/TV in femur metaphysis of aged WT females compared to young controls (*p* < 0.05). In a comparison of genotypes, aged female WT mice presented a significantly reduced BV/TV in femur metaphysis in comparison to aged 5LOKO female mice. Major differences were also found in trabecular parameters of L5. The effect of age led to a reduced BV/TV and Tb.N in aged WT female compared to young controls. In comparison of genotypes, aged female WT mice presented a significantly reduced BV/TV and Tb.N in comparison to aged 5LOKO female mice. Both aged male and female WT mice presented an increased Tb.Sp compared to 5LOKO controls. In general, WT mice were most influenced by aging compared to 5LOKO (Table 1).

### Targeted Lipidomics of Skeletal Muscle

In order to analyze the endogenous quantities of eicosanoid lipid mediators relevant in the inflammatory process of naïve skeletal muscle, we determined the concentration (pg/mg) of PGE_2_, 11,12-EET, AA, EPA, and DHA (Table 2). Considering the effect of age, WT mice presented a decrease in levels of DHA compared to their young controls, while aged 5LOKO mice presented an increase in this LM when compared with their young controls. Aged WT female mice presented an increase in levels of 11,12-EET compared to young WT female mice and compared to aged WT male mice. Considering the effect of genotype, the amounts of AA, DHA, and EPA were significantly decreased in aged female WT mice compared to aged female KO mice. Levels of 11,12-EET were significantly increased in aged 5LOKO male mice compared to aged WT male mice and also compared to aged female 5LOKO mice. Aged male WT mice also had a decrease in the levels of DHA and EPA compared to aged KO mice. Also, young and aged female WT mice had a decrease in the levels of PGE_2_ comparing with 5LOKO matched controls. Considering the effect of gender, levels of PGE_2_ were significantly increased in aged female KO mice, with significant differences compared to aged male KO mice.

**Table 2:** Targeted lipidomics of skeletal muscle in WT vs. 5LOKO mice.

**Groups Lipids (pg/mg)**	**Y WT ♂**	**Y WT ♀**	**A WT ♂**	**A WT ♀**	**Y KO ♂**	**Y KO ♀**	**A KO ♂**	**A KO ♀**
AA	4,136 ± 163	4,340 ± 177	3,221 ± 193	3,125 ± 370^#^	4,652 ± 81	4,473 ± 633	4,481 ± 859	5,288 ± 613^#^
PGE_2_	10.8 ± 3.65	6.9 ± 2.26^#^	16.77 ± 2.59	15.9 ± 3.06^#^	12.23 ± 0.64^*^	20.73 ± 15.22^#^	10.9 ± 2.78^&^^*^	45.87 ± 29^#^^&^
11,12-EET	6 ± 1.37	8 ± 3.36	4 ± 0.70^#^^&^	10 ± 3.28^&^	9.90 ± 3.36^*^	5.26 ± 2.08	23.8 ± 8.03^#^^&^^*^	8.8 ± 1.66^&^
DHA	8,386 ± 847^*^	8,899 ± 769^*^	6,078 ± 586^#^^*^	7,333 ± 1,571^#^^*^	9,679 ± 643^*^	11,580 ± 2133^*^	17,006 ± 1408^#^^*^	15,659 ± 2128^#^^*^
EPA	197 ± 21	211 ± 24	189 ± 26	172 ± 29^#^	292 ± 23	325 ± 65^#^	391 ± 108	401 ± 106^#^

*Results are presented as the means (±SD) for each lipid mediator*.

In the comparison between columns, symbol

* indicates comparison between columns: the effect of age (Y vs. A) in groups of same genotype and gender (e.g., Y ♀ WT vs. A ♀ WT); symbol

# indicates the effect of different genotypes (WT vs. KO) in groups of same age and gender (e.g., Y ♀ WT vs. Y ♀ KO); and symbol

& *indicates the effect of gender (♂ vs. ♀) in the same genotype and age (e.g., Y ♀ WT vs. Y ♂ WT). Statistically significant differences are indicated between groups with equal symbols (p < 0.05). Y = 3 months; A = 18 months*.

### Development of the Surgical Protocol

**Figure 2:**
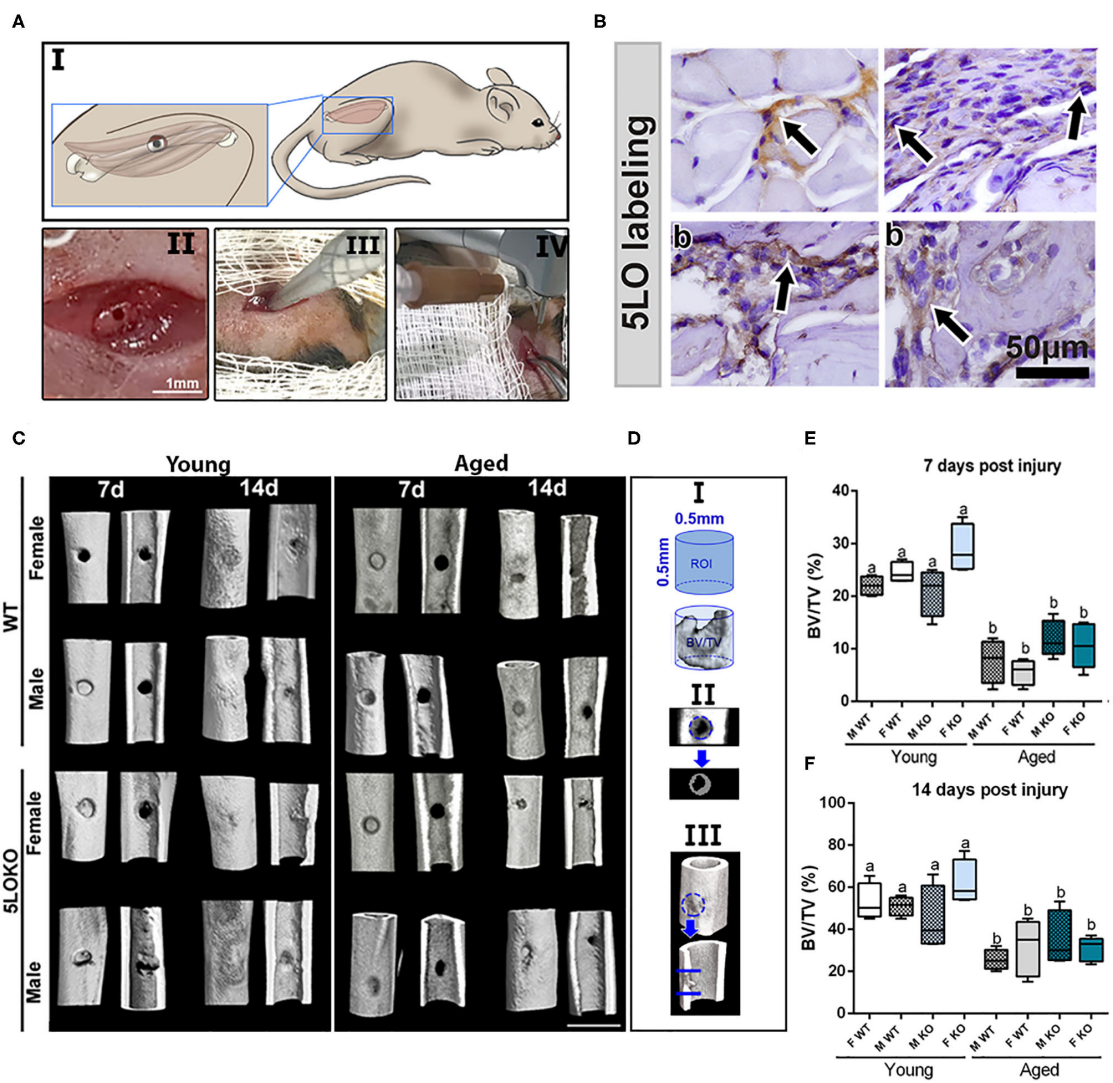
Establishment of the novel surgical bone-muscle model of simultaneous injury: **(A)** Images of the surgical model: I to IV—(I) *vastus lateralis* and femur anatomic localization in mouse and the schematic representation of muscle and bone surgical injury; (II) clinical aspect of muscle and bone injury; (III) 1-mm micro punch created a muscle defect to a depth of 1.5-mm until reaching the femoral bone; (IV) view of the subsequent defect induced by bone drilling using a 0.5-mm pilot drill at 600 rpm under constant irrigation. **(B)** Representative images for the immunolabeling of 5LO (arrows) in bone and muscle of young WT 7 days post-surgery (b), muscle (m), and periosteum (p) surrounding the bone defect. **(C–F)** MicroCT analysis: Femur containing bone defects were scanned with the microCT System (Skyscan 1174; Skyscan, Kontich, Belgium). **(C)** MicroCT 3D qualitative images were obtained from 7 to 14 days post-surgery in young and aged, male and female, and WT and 5LOKO mice. **(D)** ROI determined in the site of femur injury. **(E,F)** Bone Volume Fraction (BV/TV, %) was quantified at days 7 and 14 post-surgery, and results are presented as mean ± SD. Different letters indicate significant differences between young and aged mice for each sex and strain (*p* < 0.05).

For developing the surgical MSK injury, we created a defect in the VA muscle (1-mm in diameter) of the right lower limb, followed by a subjacent monocortical defect (0.5 mm diameter) in the midshaft of femoral bone (Figure 2A). After animal anesthesia and preparation under the stereomicroscope, the time of surgical procedure and suture was no longer than 10 min. No fractures or other additional complications were observed following the surgeries. There was no evidence of weight loss, infection, and persistent inflammation in surgical sites. The subcutaneous sutures fell off 3–4 days post-surgery when the skin was clinically healed by day 7 post-surgery. The day after the surgery, animals were able to ambulate properly and presented no major signs of distress. Importantly, of the 80 animals used in this study, two aged 5LOKO female mice died immediately after the anesthesia by accidental excess of local anesthesia combined with sedation and were replaced by two other animals. No animals were lost due to the surgical procedure and/or during the recovery phase or up to the 14-day post-surgery period.

### 5LO Is Detected in WT Mice in Bone- and Muscle-Injured Tissues

Histological slides from young male WT mice at 7 days post-surgery were used for the immunolabeling of 5LO. 5LO-positive cells were found on the cytoplasm of inflammatory cells in the granulation tissue of bone healing sites. They were also found in cells of the connective tissue of the periosteum and in the endomysium of VA muscle (Figure 2B). Results for negative immunolabeling of 5LO in 5LOKO mouse tissue are found in Supplementary Figure 1.

### Micro CT and Birefringence Analysis for Bone Healing

**Figure 3:**
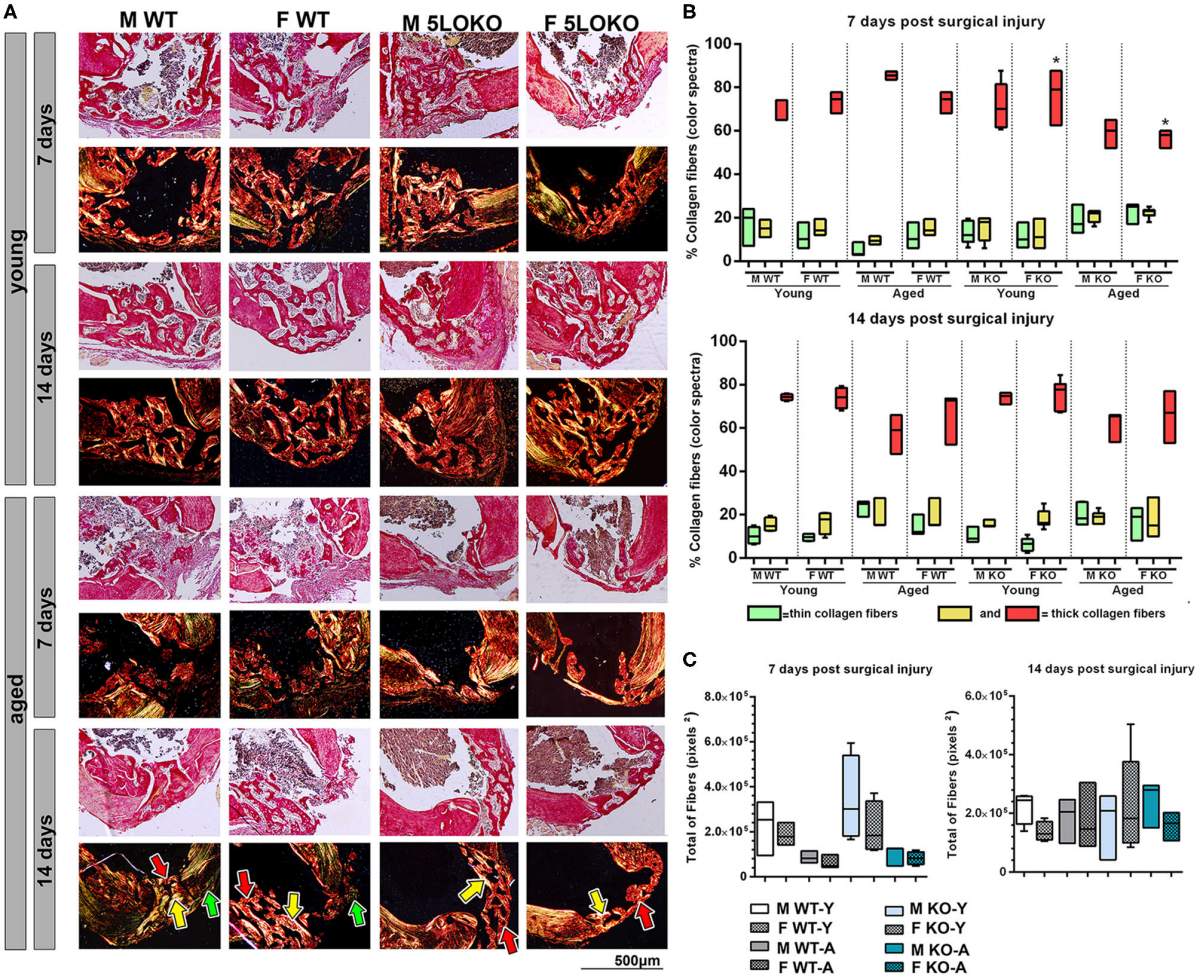
Birefringence analysis of collagen fibers during bone healing in young and aged WT and 5LOKO mice. **(A)** Representative images of the birefringence analysis are seen under polarized light. In the last panel, 14 days post-surgery of aged animals, green, yellow and red fibers bundles are exemplified by arrows in each respective color. Green birefringence color indicates thin fibers, while yellow and red colors indicate thicker collagen fibers, as indicated by arrows and in the **(B)**. **(B)** Proportion of different thickness of collagen fibers (color spectrum) considering the total of collagen in each time point post-surgery (7 and 14 days post-surgery). Slides stained with Picrosirius red and captured at 10× were analyzed in Image-analysis software (AxioVision, v. 4.8, CarlZeiss). **(C)** Total area of collagen fibers (pixel^2^) considering the sum of each spectrum of birefringence. Results are presented as mean and SD of pixels^2^ for each color in the birefringence analysis. Symbol * indicates statistically significant difference between young female KO and aged female KO (*p* < 0.05).

The proportion of mineralized bone was measured in sites of bone defect by Micro CT (Figures 2C–F). The effect of age was observed in bone healing of all groups at 7 and 14 days post-surgery, with reduced BV/TV (%) in the site of bone defects (Figures 2D,E). No significant differences were found for genders and genotypes. Considering the quality and quantity of the new collagen fibers in sites of bone healing, aged female 5LOKO mice presented a significant increase in red spectra fibers 7 days post-surgery compared to young controls (Figures 3A,B). After 14 days post-injury, no significant differences were detected in the comparison among both young groups and aged groups at the same time point (Figures 3A,B). Despite the difference pointed out at day 7 post-surgery in relation to the red fibers, total collagenous content did not show any significant differences among the groups in both time points (Figure 3C).

### Histopathological Analysis for Bone Healing

**Figure 4:**
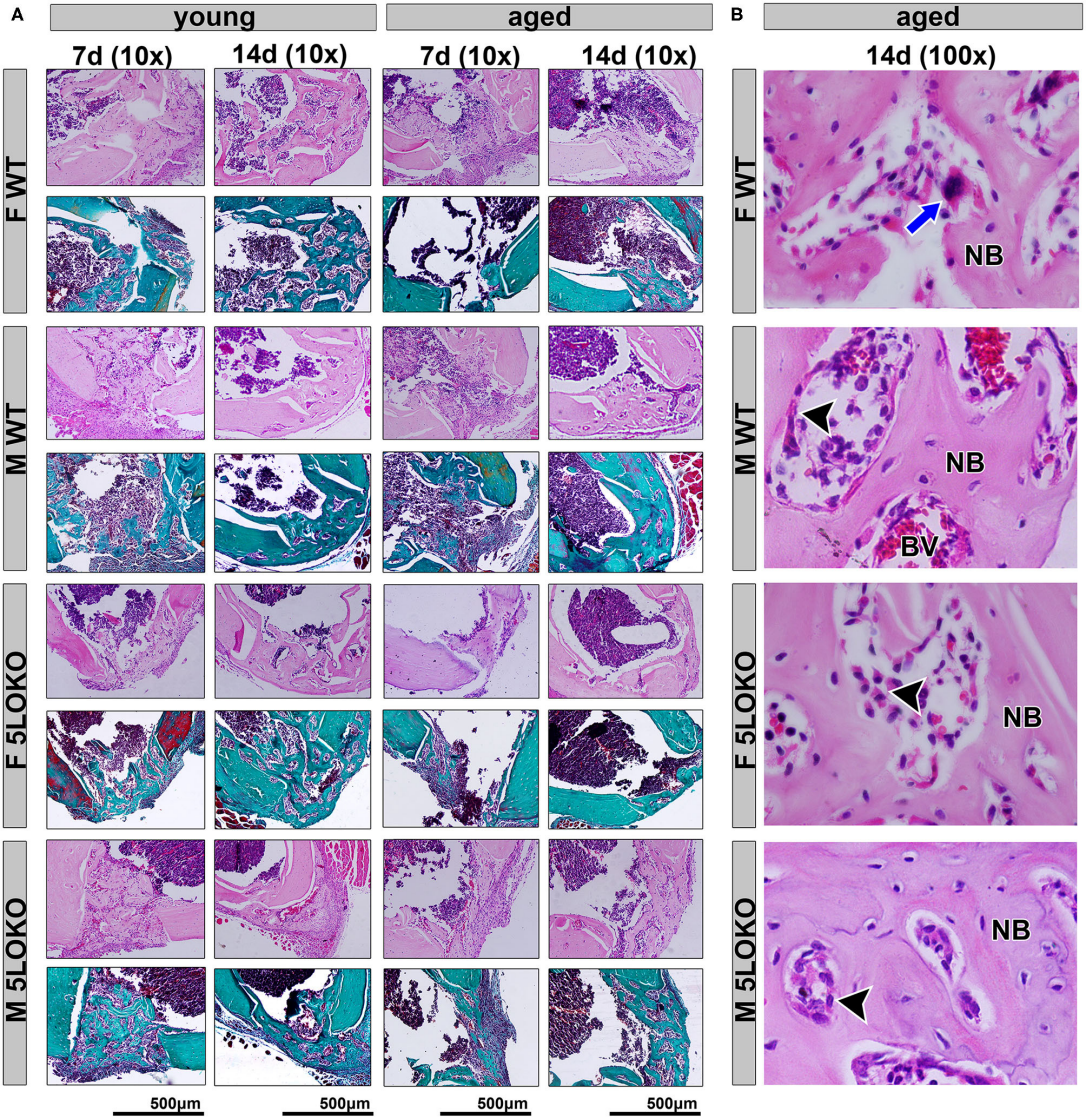
Histopathological characterization of bone healing 7 and 14 days post-surgery: Male and female, young (3 months old), and aged (18 months old) 129/SvEv WT and 5LOKO mice underwent our surgical injury model, and bone specimens were evaluated at 7 and 14 days post-surgery. **(A)** Representative images from young and aged animals (WT and 5LOKO) stained with H&E and Goldner's Trichrome/Alcian Blue. Images were captured at 10× magnification. **(B)** H&E representative images from aged animals at 14 days were captured at 100× magnification. Newly formed bone (NB), Osteoclast (blue arrows), Osteoblast (arrowhead), Blood Vessel (BV).

Our histopathological analyses showed that the bone injury site had been filled with woven bone in young female WT mice at day 7 post-surgery, characterized by a maturing process at day 14 post-surgery with osteoclastic resorption of the primary trabeculae and osteoblastic deposition of lamellar bone. In contrast, the aged female WT showed an intense leukocyte infiltration of granulation tissue in the bone defect area at day 7 post-surgery and the presence of primary bone at day 14 post-surgery, predominantly located at the defect walls. The effect of age was also observed in male WT mice. Young male WT mice presented with a similar healing process compared to the young female mice considering the same time points, while in the aged male WT mice, the defect was almost closed by maturing bone at day 14 post-surgery. In 5LOKO animals, female young mice presented a similar healing pattern to their male counterparts 7 days post-surgery, when the defect was filled with primary trabecular bone, and at day 14 post-surgery, when an advanced maturing process was observed by the presence of mature lamellar bone, similarly to the WT genotype. We noted that aged female 5LOKO mice healed the defect with a thin layer of maturing bone filling the bone defect at day 14 post-surgery, while in the aged 5LOKO male mice, it was mostly filled with mature trabecular bone. Comparing the genotypes, we observed major protective effects in aged female 5LOKO compared to aged WT mice, since aged 5LOKO presented an improved bone healing compared to aged WT females, with less inflammation at day 7 post-surgery a more mature bone at day 14 post-surgery in 5LOKO females (Figure 4).

### Histopathological and Histomorphometric Analysis for Skeletal Muscle Healing

**Figure 5:**
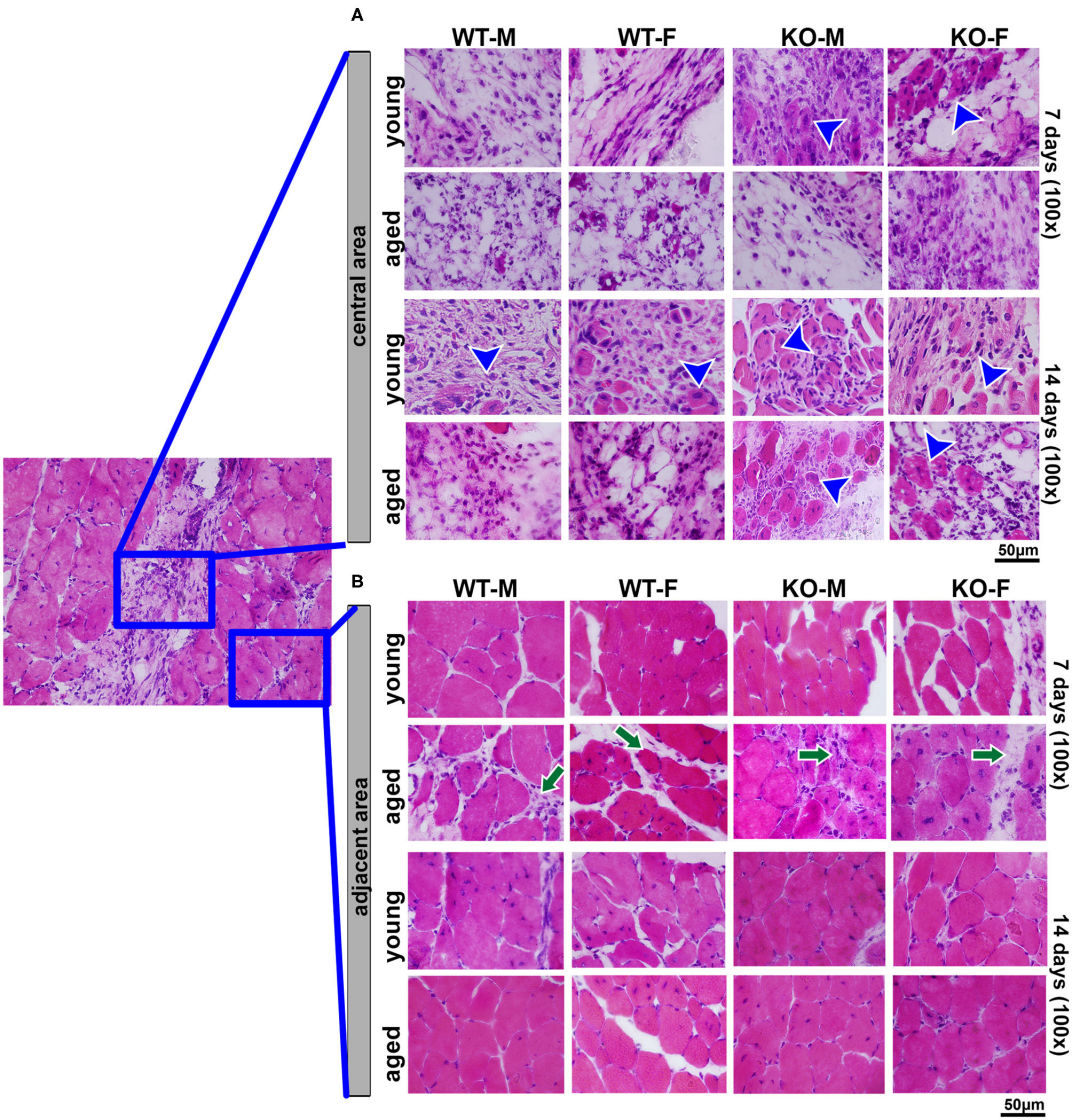
Histopathological analysis during muscle healing in young and aged WT and 5LOKO mice: Male and female, young (3 months old), and aged (18 months old) 129/SvEv WT and 5LOKO mice underwent simultaneous surgical muscle and bone injury, and bone specimens were evaluated at days 7 and 14 post-surgery. **(A)** Representative images from the central area of muscle injury. Blue arrowheads indicate centralized myonuclei in the region of damage. **(B)** Representative images from the adjacent area of injury. Histological transversal sections were stained with H&E. Images were captured at 100× magnification. Green arrows indicate connective tissue among muscle fibers.

The effects of age were most detrimental regarding the histopathological description of aged WT mice, compared to the controls in both genders. At day 7 post-surgery, a key observation in the site of muscle injury of young WT male mice was the presence of loose connective tissue with eventual mononuclear leukocytes, while aged male WT mice showed a mixture of connective and adipose tissue infiltrated by neutrophils. The same pattern was observed in the group of WT female animals, except that at day 7 post-surgery, the young female mice presented dense connective tissue at the site of muscle injury. In the comparison of different genotypes, 5LOKO young animals, both male and female, showed clear differences as compared to WT mice especially at day 7 post-surgery, when muscle cells with centralized myonuclei could be seen surrounded by connective tissue. However, both male and female aged 5LOKO mice exhibited an injury site filled by connective tissue. Only at day 14 post-surgery, some muscle cells were observed in the injury site in young male and female WT mice, while in the aged mice the site of injury showed a disorganized connective tissue with adipose cells and focal mononuclear and neutrophil leukocytes infiltrate. Nonetheless, at the site of muscle injury in 5LOKO young males, numerous muscle fibers with centralized myonuclei could be seen at day 14 post-surgery. The key difference we found in the site of injury in the aged male 5LOKO animals was that a higher amount of connective tissue could be observed surrounding the muscle fibers. At this same period, female 5LOKO young mice also showed some muscle fibers with centralized myonuclei, but connective tissue was predominant. Muscle fibers with centralized myonuclei were also seen in the defect of 5LOKO aged female mice, but a loose connective tissue highly infiltrated by neutrophils was noted surrounding the muscle fibers (Figure 5A). Non-inflammed connective tissue was also observed among muscle fibers in adjacent areas of the injured sites of both WT and 5LOKO female and male aged mice (Figure 5B).

From the histomorphometric analysis, the percentage of muscle inflammation did not present significant differences at day 7 post-surgery, but was significantly increased in all aged animals at day 14 post-surgery, evidencing the detrimental effect of aging in muscle healing of both genotypes and genders. When comparing the 5LOKO male and female groups with their matched WT strains, it was detected that muscle inflammation decreased in both male and female aged 5LOKO at day 14 post-surgery. We also determined that aged female WT mice was the only group that showed a significant decrease in muscle fiber CSA when compared to their young WT group at day 14 post-surgery. We also identified an increase in the number of centralized myonuclei in aged 5KOLO female mice between day 7 and 14 post-surgery (Table 3).

**Table 3:** Histomorphometry of injured muscles in WT vs. 5LOKO mice.

**Parameters**	**Groups/Periods**	**Y WT ♂**	**Y WT ♀**	**A WT ♂**	**A WT ♀**	**Y KO ♂**	**Y KO ♀**	**A KO ♂**	**A KO ♀**
Muscle Inflam (%)	7 days	16 ± 5^a^	14 ± 2^a^	14 ± 5^a^	15 ± 10^a^	11 ± 3^a^	13 ± 4^a^	16 ± 4^a^	14 ± 7^a^
	14 days	14 ± 6^a^^*^	18 ± 9^a^^*^	43 ± 8^b^^*^^#^	38 ± 7^b^^*^^#^	10 ± 4^a^^*^	11 ± 5^a^^*^	25 ± 7^b^^*^^#^	22 ± 4^b^^*^^#^
CSA (μm^2^)	7 days	1,088 ± 661^a^	977 ± 474^a^	894 ± 515^a^	955 ± 424^a^	1,341 ± 715^a^	1021 ± 516^a^	994 ± 199^a^	751 ± 290^a^
	14 days	1,298 ± 724^a^	1,127 ± 446^a^^*^	739 ± 94^a^	505 ± 101^a^^*^	1,451 ± 432^a^	1,361 ± 530^a^	869 ± 383^a^	741 ± 319^a^
Centralized Myonuclei (%)	7 days	24 ± 18^a^	26 ± 20^a^	20 ± 11^a^	19 ± 10^a^	37 ± 13^a^	34 ± 8^a^	26 ± 4^a^	24 ± 10^a^
	14 days	23 ± 16^a^	28 ± 17^a^	21 ± 18^a^	25 ± 11^a^	39 ± 17^a^	37 ± 14^a^	29 ± 15^a^	37 ± 12^b^

*Results are presented as the means (±SD) for each parameter*.

In the comparison between lines, different letters (a vs. b) indicate significant difference for time points (7 vs. 14 days post-surgery) of the same group in each evaluated parameter (p < 0.05). In the comparison between columns, symbol

* indicates comparison between columns: the effect of age (Y vs. A) in groups of same genotype and gender (e.g., Y ♀ WT vs. A ♀ WT); symbol

# *indicates the effect of different genotypes (WT vs. KO) in groups of same age and gender (e.g., Y ♀ WT vs. Y ♀ KO). Statistically significant differences are indicated between groups with equal symbols (p < 0.05). Y = 3 months; A = 18 months. No statistical significant differences were found between genders (♂ vs. ♀) in the comparison of the same genotype and age (e.g., Y ♀ WT vs. Y ♂ WT)*.

### Immunolabeling and Histomorphometric Analysis of Muscle and Bone Markers of Remodeling

**Figure 6:**
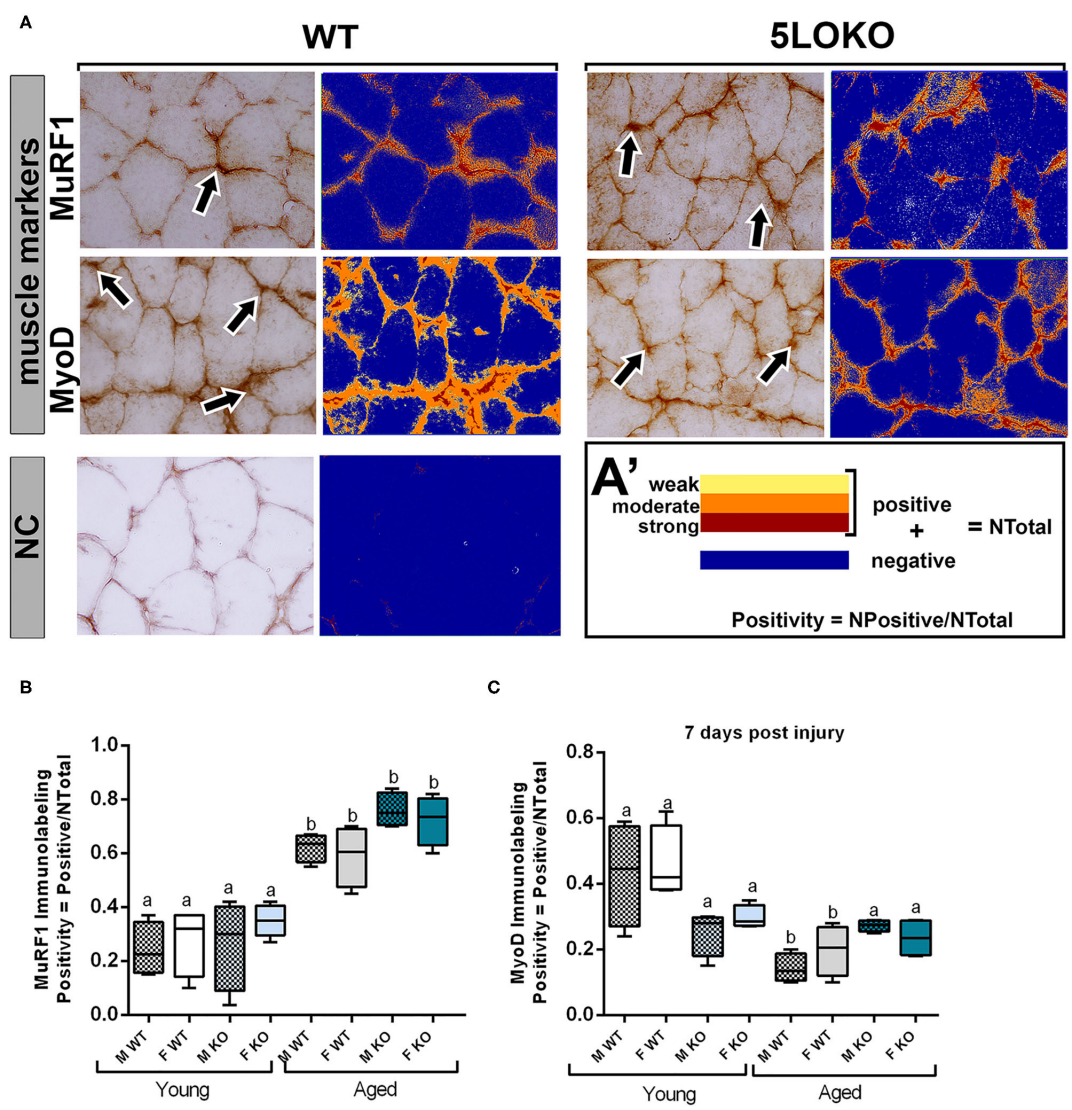
Histomorphometric characterization and Immunolabeling of MuRF1 and MyoD in WT and 5LOKO mice: **(A)** Representative images for MuRF1 immunostaining and MyoD immunostaining (arrows) in male young SvEv WT mice at 7 days post-surgery. DAB was used for the antigen–antibody reaction. Muscle samples were left without counterstaining to be used for optical density quantification. **(A–A′)** Positive Pixel quantification was performed by the software Aperio Image Scope v/ 12.3.3 (Leica Biosystems, Buffalo Grove, USA). Images were analyzed with algorithm Positive Pixel Count version 9 and are expressed as Positivity (number of positive pixels/number total). Negative controls (NC) were used for calibration and confirmation. **(B,C)** Results from positive pixel quantification for MuRF1 and MyoD were presented as means ± SD for each marker. Different letters indicate significant differences between young (3 months old) and aged (18 months old) mice for each sex and strain (*p* < 0.05).

Immunolabeling for both MuRF1 and MyoD was performed at day 7 post-surgery and positive pixel quantification was performed. MuRF1 was predominant in muscle nuclei (Figure 6AA'). The effect of age in MuRF1 immunolabeling (e.g., young male WT vs. aged male WT) was observed in both studied strains and genders, with a significant decrease in these markers for all aged animals (Figure 6B). No significant differences were obtained for MuRF1 among the genotypes and genders (Figure 6B). For MyoD, aged male and female WT mice showed a decreased immunolabeling for MyoD when compared to the matched gender young mice, but no effects of age were observed in 5LOKO mice (Figure 6C). No significant differences were found considering the effect of gender and genotype.

**Figure 7:**
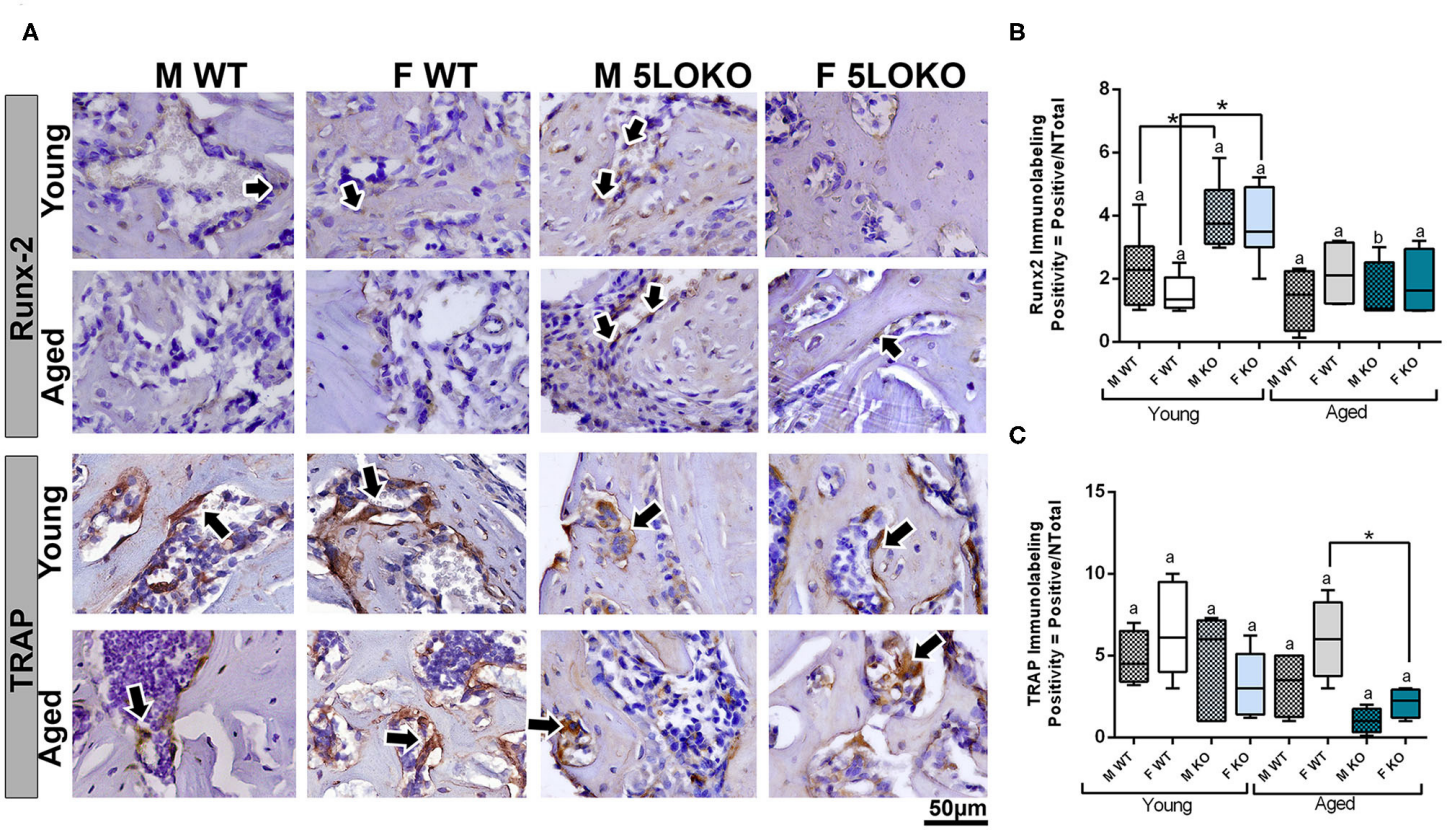
Histomorphometric characterization and Immunolabeling of Runx-2 and TRAP in WT and 5LOKO mice: **(A)** Representative images for Runx2 immunostaining and TRAP immunostaining (arrows) of male and female, young (3 months old), and aged (18 months old) 129/SvEv WT and 5LOKO mice. Identification of antigen–antibody reaction was performed using DAB, counterstained with Mayer's hematoxylin. **(B,C)** Pixels quantification of Runx2 **(B)** and TRAP **(C)** was performed by the software Aperio Image Scope v/ 12.3.3 (Leica Biosystems, Buffalo Grove, USA). Images were analyzed with algorithm Positive Pixel Count version 9 and are expressed as positivity (umber of positive pixels/number total). Negative controls were used for calibration and confirmation. Results were presented as the mean and SD for each marker. Different letters indicate significant differences in between young (3 months old) and aged (18 months old) mice for each sex and strain (*p* < 0.05); symbol * indicates significant differences between WT vs. 5LOKO at the same age.

Dynamics of bone modeling/remodeling in bone-injured sites were evaluated with anti-Runx-2 and anti-TRAP antibodies (Figure 7A). Considering the effect of age for Runx-2, a significant decreased in the area density of positive cells were found in aged 5LOKO male mice compared to young controls. Considering the effect of genotype, both male and female 5LOKO young mice demonstrated an increase in the area density of Runx-2+ cells compared to their matched WT controls at day 7 post-surgery (Figure 7B). TRAP immunolabeling was significantly increased in the bone damage sites of female aged WT animals when compared to 5LOKO mice at day 14 post-surgery (Figures 7A,C).

## Discussion

Aging and inflammation can negatively affect the MSK system of male and females in a different manner and proportion (12, 64, 65). In our results for bone phenotyping, we found significant differences in macroscopic features comparing genders, with higher values (BW and/or RC length) for males compared to females in both genotypes. We observed major detrimental aging effects in female WT mice, with decreased in trabecular bone parameters (BV/TV and Tb.N) in L5 vertebral body compared to aged WT males.

Considering the genotype, aged 5LOKO mice also displayed a higher size compared to aged WT, but additional studies are necessary to observe the proportion of adipose tissue and muscles in mice lacking 5LOKO. A previous study has associated the 5LO gene (also called Alox5) as a candidate gene for obesity and low bone mass when 5LOKO (in the C57Bl/6 background) is subjected under long-term treatment with high fat diet (containing 45% fat by kcal and 3.0g of AA). Briefly, in this previous study, male and female 5LOKO mice under this treatment presented decrease in Tb.N compared to WT (at final age of 16 weeks, or ~ 4 months) (66). In our comparisons by microCT, we observed an increased bone quality in young and aged 5LOKO mice, compared to WT controls. It is also important to emphasize the differences in this particular methodology: 129Sv background, different ages (4 months for young and 18 months for aged mice) and a standard diet. In agreement, a previous study using radiology and bone histomorphometry has shown that both male and female 5LOKO mice, at 7 weeks age, from either 129Sv or mixed C57Bl/6x129sv background, have increased cortical bone compared with WT controls (36). Our study suggested that 5LO knockout protects against age-related bone loss in females, since the effect of aging on 5LOKO females was attenuated and they presented an increased BV/TV and Tb.N in femur and L5 compared to aged WT controls. It is also important to emphasize the differences in this particular methodology: different ages (4 months for young and 18 months for aged mice) and a standard diet.

It has been demonstrated that females have higher 5LO activity and/or higher LT production compared to males which could impact other pathways of the eicosanoid system (46). Using a unique customized approach of targeted lipidomics in gastrocnemius muscles of young and aged C57Bl/6 mice, a previous study has demonstrated lipid signaling is age- and gender-dependent (12). Our results from targeted lipidomics in gastrocnemius muscles demonstrate that the deletion of 5LO in mice significantly impact the profiling and quantities of lipid mediators in skeletal muscles of 5LOKO mice. In accordance, the endogenous levels of AA, EPA, and DHA were significantly decreased in aged female WT mice compared to aged female 5LOKO. Levels of 11,12-EET were significantly increased in aged 5LOKO male mice compared to aged WT mice. Aged male WT mice also presented a decrease in the levels of DHA and EPA compared to aged KO mice. Also, levels of PGE_2_ were significantly increased in skeletal muscle of aged female KO mice compared to aged male KO mice. Considering the effect of gender, levels of PGE_2_ were significantly increased while 11,12-EET was decreased in aged female KO mice, with significant differences compared to aged male KO mice. It is tempting to postulate that such combination of an elevation of PGE_2_, which was demonstrated at nanomolar levels to enhance myogenic differentiation of C2C12 mouse muscle cells and increase *ex vivo* contractile force (3, 4), and the decreased levels in 11,12-EET could combine in the female 5LOKO to promote improved MSK healing. Although AA-derived lipid mediators are known to play a major pro-inflammatory role, PGE_2_ can have a dual role, modulating both pro- and anti-inflammatory responses (67). Furthermore, PGE_2_ can accelerate myogenesis differentiation (4) and induce osteogenesis (27, 30, 32). EPA and DHA are involved in the lipid-mediator profile switching during resolution of inflammation. In this context, EPA-derived mediators have a low pro-inflammatory potential, whereas DHA is a major source for the final anti-inflammatory products D-series resolvins and protectins required for inflammation resolution without fibrosis (68–70). EET acids, such as 11,12-EET, are generated by the activity of cytochrome p450 (CYP) enzymes on AA (71). 11,12-EET can play a modulatory role on COX-2 activity and attenuate the synthesis of PGE_2_ during LPS-induced inflammatory response in rat monocytes (72). Other previous studies have shown that 11,12-EET can play anti-inflammatory activities by inhibition TNFα-induced VCAM-1, E-selectin, and ICAM-1 expression in endothelial cells (73). In our results, aged 5LOKO females presented an increased amount of PGE_2_ compared to aged WT females, and conversely, aged 5LOKO females presented a decreased amount of 11,12-EET. An important consideration about the effects of LMs, myokines, osteokines, and secreted factors from bone-muscle and tissues in general is that we should apply accepted knowledge from endocrinology to this growing and fertile field of investigation.

To investigate the role of 5LO in the process of simultaneous bone and muscle healing, we developed a new surgical model of MSK injury in 5LOKO animals. Despite the importance of *in vivo* investigation for the comprehension of bone healing, previous rodent models of bone and muscle damage focus their attention exclusively on bone formation (14, 74), while the majority of studies of the on MSK healing addresses skeletal muscle (23, 75) or bone, but separately (24, 39). In our study, we performed a moderated simultaneous bone and muscle surgical damage, with a 1-mm diameter defect in muscle and a 0.5-mm diameter defect in the subjacent bone (Figure 2A), in order to avoid further surgical complications, such as femur fracture in aged animals. Our primary goal was to mirror human conditions of simultaneous tissue damage where recovery is feasible and to avoid any type of major catastrophic failure. Our results revealed a different pattern and timing in bone and muscle healing in WT mice. Interestingly, the bone defects of young male and female mice were completely filled with woven bone by day 7 post-surgery, and maturing by day 14 post-surgery, as previously demonstrated for bone healing in the tibia of mice, but in a non-simultaneous muscle defect model (76). On the contrary, muscle healing is clearly delayed in the same animals. In the site of muscle injury of young male and female WT mice there was still connective tissue with eventual mononuclear leukocytes at 7 days post-surgery, with no significant changes in area density of inflammatory infiltrate at 14 days post-surgery. It is important to mention that bone has significant capacity for remodeling/regeneration regarding bone lining cells and/or osteoprogenitors lineages as stable cells, which are found in quiescent stage in physiologic conditions, but can undergo rapid proliferation in response to an injury (20). On the other hand, muscle fibers rapidly undergo degeneration after extensive damage (77, 78), and damaged muscle cells can still be found after 6 days in mouse models of surgical injury (17). Simultaneously and in response to the mediators released to the site of injury, satellite cells can proliferate and differentiate in new myotubes followed by upregulation of MyoD (7). This early process was observed during the first 7 days in mouse models of muscle injury (17, 78). MuRF1 was used in this study as a marker for fiber degeneration as a consequence of the surgical injury, as previously described (79), while MyoD was used as a marker of new fibers. MyoD induces the expression of muscle-specific genes in myoblasts and rapid cell proliferation in models of crush-induced injury, but it may be undetectable in newly formed myotubes (17, 18). We detected both MuRF1 and MyoD 7 days post-injury. An intriguing possibility is that both the degradation of muscle cells and the burst in satellite cell activity and myoblast proliferation could lead to a surge of myokines near the bone damage, which in turn helps bone to accelerate regeneration, and it occurs with muscle flaps, the skeletal muscle functions to assist in the faster recovery of bone tissue.

Other previous models of combined bone and muscle injury have been done using models of volumetric muscle loss injury, and they are more common rats. Usually combined bone and muscle injury models in rodents involve volumetric muscle-loss injuries, followed by endogenously healing osteotomy (80) or severe open fractures (81–83) and non-endogenously healing femur defects (84). Due to the nature of trauma and embryologic origin of femur, bone fractures generally heal throughout a cartilaginous callus formation, previous to woven bone deposition (80), while in our mouse model the bone healing was noted to be predominantly driven by intramembranous healing. Together, these previous studies have also evidenced impaired bone healing related with concomitant muscle trauma and increased levels of inflammatory markers in sites of injury combined, compared to isolated injuries (80). When muscles are severely traumatized, the sustained inflammation diminishes mechanical bone strength and decreases mineralized matrix in the site of injury (81). Interestingly, these previous studies evaluated bone and muscle defects with or without combination, to permit additional comparisons and better determine the role of muscle in bone healing and *vice versa*. Comparatively, in our study we only produced combined injuries for primary study due the limited number of animals for studying 3 variables (two ages, genders, and genotypes). Additionally, our model does not aim to challenge bone and muscle healing with critical injuries, and it is performed in a very controlled manner, in order to avoid complications related to the surgical procedure. In this way, it is possible to investigate the MKS healing in compromised and fragile animals, such as aged mice.

Considering the advantages of our animal model, we decided by developing this combined defects in mice, considering a number of advantages and the cost–benefit related to mice when comparing with other rodents, such as the possibility to explore genetic approaches (e.g., 5LOKO mouse model utilized in this study); the reduced size compared to rats, which consequently reduces quantities of experimental drugs; and reduced experimental periods (85). In the context of bone–muscle crosstalk field, mouse is a suitable animal model for pharmacological and genetic interventions. Also, while timing of muscle and bone healing ranges from 4 to 12 weeks in rats, this type of MSK injury in mice was evaluated along 14 days. Even including longer time points, it might not exceed 4 weeks due the accelerated mice metabolism compared to other species. Comparing our model with preclinical orthopedic mice models (e.g., closed and open fractures), our study provides a surgical model that can be consistently reproduced with a rapid and simple surgery, minimal timing consuming, improving the animal recovery and avoid the risk of infections (86). On the other hand, since it is a non-critical muscle and bone defect, the present model does not challenge beyond physiological bone-muscle healing mechanisms in non-compromised animals (e.g., young adult and/or health). This is a major advantage in the context of normal aging.

During aging, the MSK system shows a decreased capacity for healing, associated with increased levels of inflammation (7, 11, 87). Our results revealed a significant impairment on both bone and muscle healing in aged mice when compared to the young controls. The fraction of newly formed (BV/TV, %) bone analyzed by microCT was significantly decreased in all aged mice, considering both genotypes and genders (Figures 2C–F). Despite increased amount of thicker collagen fibers in young 5LOKO females compared to aged controls, no major differences were found in the collagen content of bone matrix (Figure 3A). In skeletal muscles, a significant increase in inflammatory infiltrated in the VL muscles was noted at day 7 and 14 post-surgery (Table 3). CSA was also significantly decreased in aged WT females compared to the young controls, while aged 5LOKO females maintained the CSA compared to young 5LOKO females (Table 3). Additionally, while MuRF1 immunolabeling was increased in male and female aged mice (both genotypes), decreased immunolabeling for MyoD was observed to the matched young groups (for WT genotype), suggesting a reduced capacity for remodeling and regeneration along aging (Figure 6). These observations confirm a detrimental effect of aging in all groups, but with the attenuated effect of the aging effect in 5LOKO mice, especially in histopathological comparisons between aged WT vs. aged 5LOKO females.

After 7 days of surgical trauma, injured muscle, periosteum, and bone of WT mice were assessed for 5LO expression (Figure 2B). In support of 5LO roles on inflammation (25), 5LO positive cells were predominantly found in the leucocytes infiltrating bone and muscle healing sites, but also were found in the constitutive cells of connective tissue from the periosteum. In agreement with our findings, a mouse model of femur closed-fracture healing has demonstrated strong positive immunolabeling of 5LO in bone marrow leukocytes, periosteum, osteoclast, muscle interstitial cells, and chondrocytes until 7 days post-injury (27). Given the limitations of immunohistochemistry techniques, future molecular investigation is necessary to explore 5LO in injured and non-injured bone and muscle cells by other approaches.

No previous studies have addressed the effects of 5LO or its metabolites in combined MSK injuries. We next evaluated the influence of 5LO on bone and muscle healing in different ages and gender, by comparing WT vs. 5LOKO. Isolated evaluation of bone injury using bone fracture healing models have previously demonstrated that genetic deletion of 5LO in mice (28) or its pharmacological inhibition (39, 40) accelerate fracture callus formation in young male rodents. An important finding in our studies was that young female and male 5LOKO mice presented significantly increased Runx-2 expression in comparison with matched WT animals at day 7 post-injury, but with no significant differences in BV/TV of newly formed bone. Additionally, genetic deletion of 5LO in females led to attenuated inflammatory response after the simultaneous injury. While an intense inflammatory response in the bone-injury site of aged WT females 7 days post-injury with a delay in healing (14 days) was clear, aged 5LOKO presented less inflammatory infiltration and earlier mature bone (7 days) (Figure 4). TRAP-positive osteoclasts were also increased in aged females WT compared to 5LOKO at day 14 post-surgery (Figure 7), in agreement with the role of LTs on osteoclast formation in bone resorption and inflamed environments (34, 35). These results suggest that the 5LO signaling pathway may play a negative role on bone healing. Furthermore, young 5LOKO mice displayed advanced healing in VL muscle defects compared to WT mice, with centralized myonuclei surrounded by connective tissue while highly inflamed muscles could be seen in WT animals. Both male and female aged 5LOKO mice exhibited an injury site filled by connective tissue, while aged controls presented connective and adipose tissue (Figure 4). Interestingly, the number of positive MyoD cells was already decreased in young 5LOKO mice 7 days post-injury compared to WT (Figure 6), but the 5LOKO presented a higher amount of centralized myonuclei indicative of muscle regeneration by day 14 post-surgery (Table 3). Furthermore, MyoD positive cells were decreased in aged WT, compared to the young WT mice, but not in aged 5LOKO mice compared to young 5LOKO mice (Figure 6). Future studies could conduct longitudinal studies to help further clarify the roles of 5LO in modulating the levels of muscle regulatory proteins.

**Figure 8:**
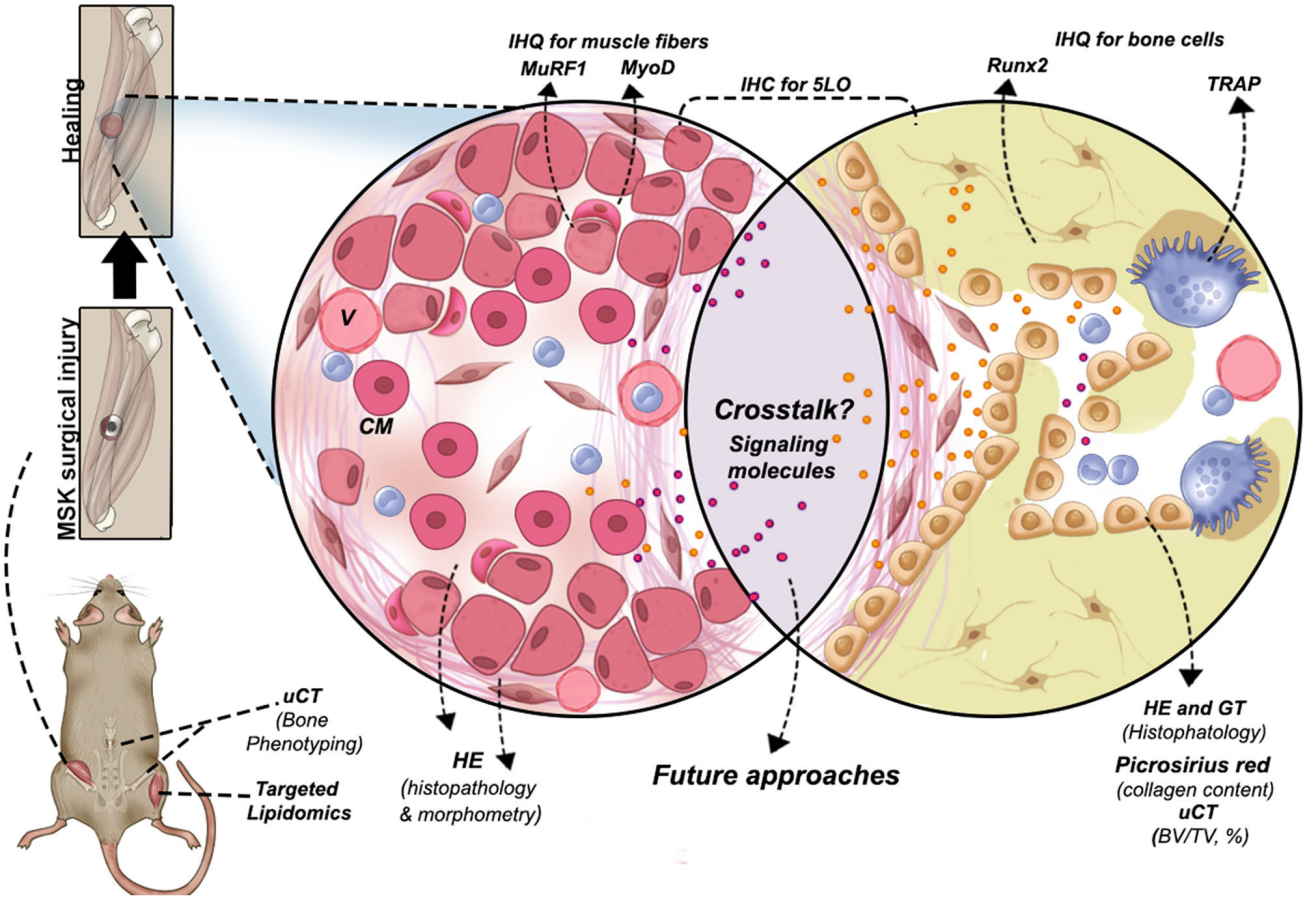
Graphical abstract of the new surgical protocol for MSK combined injury and methodologies performed to evaluate bone-muscle injury in mice. Other possibilities are suggested to investigate signaling molecules involved in bone and muscle crosstalk during tissue regeneration. CM, centralized myonuclei; V, blood vessels.

In summary, our results demonstrate that 5LOKO mice present important differences in bone phenotyping and lipid profiling and quantification in skeletal muscles. Notably, the deletion of 5LO plays a protective role in bone of aged female 5LOKO mice and significantly impacts the capacity of MSK healing during aging. It is important to emphasize that this study presents some limitations, including detailed functional approaches and molecular quantification of 5LO and its products in both age and genders. However, our detailed lipidomic analyses, along with the bone phenotyping, allowed us to we hypothesize that changes found in skeletal muscle levels of LMs (particularly the intriguing aforementioned combination of elevation of PGE_2_ and reduction of 11,12-EET) in 5LOKO may contribute to explain the improved MSK healing in these mice. Reinforcing this hypothesis, aged 5LOKO females mice presented increased amount of anti-inflammatory lipids (EPA and DHA) in comparison to aged WT females. We propose that future studies could investigate the levels of LMs in injured sites as well as expand the investigation on the role of 5LO in the MSK system, by using pharmacological and molecular-genetic approaches. The new model of simultaneous bone-muscle injury is pathophysiologically relevant for future studies in bone–muscle crosstalk, as well as suitable to advance the understanding of MSK healing during aging (Figure 8). We finally suggest the 5LO signaling pathway as a potential target for interventions against post-menopausal osteoporosis.

## Conclusion

This study described a suitable mice model of simultaneous bone–muscle healing in order to contribute to the investigation of future bone–muscle crosstalk studies (Figure 8), revealing that simultaneous bone–muscle healing was influenced by aging, gender, and the 5LO pathway. In general, 5LOKO aged female mice presented improved healing capacity when compared to their matched WT groups. Our lipidomic analyses elicited intriguing possibilities for the roles of lipid signaling mediators in skeletal aging and MSK healing. Our studies suggest that 5LO plays a crucial role in both modulation and resolution of inflammation during aging.

## Data Availability Statement

The raw data supporting the conclusions of this article will be made available by the authors, without undue reservation.

## Ethics Statement

The animal study was reviewed and approved by Ethic Committee on Animal Use of the Sagrado Coração University (CEUA/USC).

## Author Contributions

CB and MM contributed to the conception and design, the acquisition, analysis, and interpretation, drafted the manuscript, critically revised the manuscript, gave final approval, and agreed to be accountable for all aspects of work. MB contributed to the analysis and interpretation, drafted the manuscript, critically revised the manuscript, gave final approval, and agreed to be accountable for all aspects of work. MC, ACS, JS, VR, ALS, and ZW contributed to the acquisition, analysis, and interpretation of the manuscript, critically revised the manuscript, gave final approval, and agreed to be accountable for all aspects of work. JA contributed to the conception and design, the interpretation and critically revised the manuscript, gave final approval, and agreed to be accountable for all aspects of work. All authors contributed to the article and approved the submitted version.

## Conflict of Interest

The authors declare that the research was conducted in the absence of any commercial or financial relationships that could be construed as a potential conflict of interest.
