# Supplementary material for: New Surgical Model for Bone–Muscle Injury Reveals Age and Gender-Related Healing Patterns in the 5 Lipoxygenase (5LO) Knockout Mouse
Source: Front Endocrinol (Lausanne). 2020 Aug 11;11:484. doi: 10.3389/fendo.2020.00484 (PMC7431610; doi:10.3389/fendo.2020.00484)
Supplement: Supplementary file 1 [file Data_Sheet_1.docx]

Supplementary Material


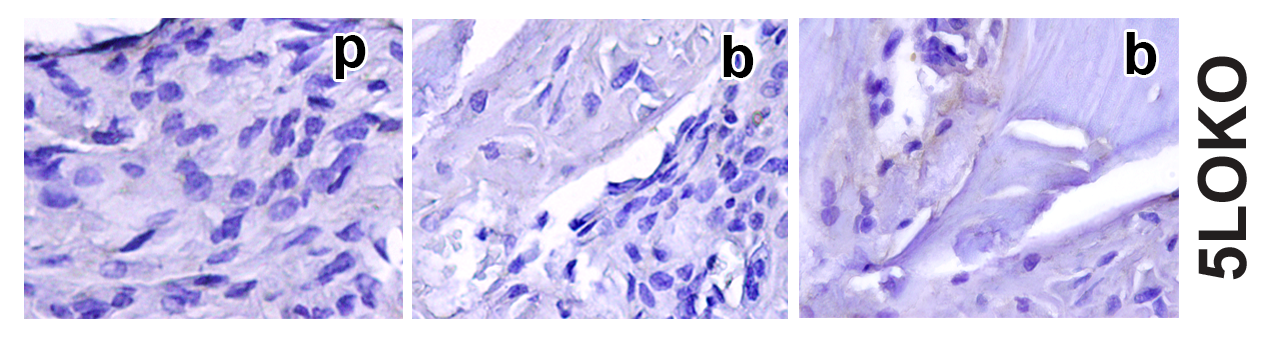


**Supplementary Figure 1.** Representative images for the immunolabeling of 5LO injured sites of young 5LOKO mice at 7 days post-injury); (b) bone and periosteum (p) surrounding the bone defect.
